# Food Insecurity Among Post-Secondary Students in High Income Countries: Systematic Review and Meta-Analysis

**DOI:** 10.1007/s13668-025-00651-2

**Published:** 2025-04-08

**Authors:** Fiona H. McKay, Bolanle Racheal Olajide, Lisa J. Melleuish, Penelope Pitt, Eric HY Lau, Matthew Dunn

**Affiliations:** 1 School of Health and Social Development, Faculty of Health, Deakin University, Geelong, VIC 3220 Australia; 2 Institute for Health Transformation, Faculty of Health, Deakin University, Geelong, VIC 3220 Australia; 3 Deakin University Student Association (DUSA), Deakin University, Burwood, Australia; 4 School of Public Health, Li Ka Shing Faculty of Medicine, University of Hong Kong, Hong Kong, China

## Abstract

**Purpose of Review:**

Food insecurity has recently emerged as a growing problem among students attending post-secondary institutions in high income countries, where it is associated with poorer health outcomes and poorer academic performance. The aim of this systematic literature review and meta-analysis is to synthesize evidence from published studies describing the prevalence of food insecurity among students in post-secondary educational institutions. A broad search was employed to identify any studies related to food security among post-secondary students in high income countries (PROSPERO identifier: CRD42023468944). Four electronic databases were systematically searched: Medline, CINAHL, Global Health, and Embase, in November 2023. Key thematic areas searched were food insecurity and education; no temporal limitations were placed on the search. Only English language peer-reviewed articles were considered. Four authors independently reviewed all articles to identify relevant studies.

**Recent Findings:**

156 studies are included in this review. Sample sizes ranged from 10 to 122,269 participants (total participants 743,075; 409,047 women) with a median of 4764. Most studies were based in the USA (*n* = 139, 88%), and most were cross-sectional (*n* = 131, 84%). All articles were published after 2009, with more than three-quarters (*n* = 121, 77.5%) published after 2020. One hundred (64%) studies reported the mean age of participants; across these studies the average age was 22.4 years (range 18 to 78 years). Almost all studies employed one form of the USDA household food security survey module. Food insecurity across the whole sample ranged from 11.8 to 98%, the pooled estimate for food insecurity was 42.2%, (95% CI = 38.8–45.8%).

**Summary:**

Our findings show that a high proportion of students attending post-secondary institutions experience food insecurity, potentially leading to both poorer academic and health outcomes.

**Supplementary Information:**

The online version contains supplementary material available at 10.1007/s13668-025-00651-2.

## Introduction

Food security exists when all people, at all times, have physical and economic access to sufficient, safe, and nutritious food that meets their dietary needs and food preferences for an active and healthy life [1]. Food insecurity is the inability to maintain or acquire adequate food, which may result in disrupted eating patterns and decreased nutrient intake [1], and can be the result of a lack of financial or other resources, or a problem with the food system [2, 3]. Hunger is the most serious outcome of food insecurity and typically refers to insufficient consumption of food or energy required for daily living and activity [4]. Food insecurity impacts millions of people globally, and while there has been a significant and important focus on food insecurity in low- and middle-income countries, there is an increasing recognition that food insecurity also exists in high income countries. Despite increased access to resources and economic advantages, approximately 10% of people living in high-income countries experience food insecurity [5], with factors relating to food insecurity varying by level of development and Gross Domestic Product per capita [6].

Food insecurity is a social determinant of health and can lead to and result from a range of household stressors. Some groups are more likely to be impacted by food insecurity; this includes households with children [7, 8], people on low incomes or people who are unemployed [9], and people living in remote or rural areas [10, 11]. Recently, food insecurity has emerged as a growing problem among students attending post-secondary institutions, including universities, colleges, and other tertiary institutions (from herein referred to collectively as post-secondary institutions). Research has explored the factors that impact food insecurity among post-secondary students, finding that students from ethnic minority groups and undergraduate students are more likely to experience food insecurity than other students [12, 13]. Students who are financially independent on their parents and in receipt of financial assistance such as scholarships or student loans are also at higher risk of experiencing food insecurity [14]. Student food insecurity is associated with poor health outcomes including overall self-rated health [15, 16], poor mental health [17], and poorer academic outcomes [18].

Responding to student food insecurity and hunger requires further understanding of the prevalence and impacts of food insecurity among students attending post-secondary institutions. There are several previous reviews that have explored food insecurity in post-secondary settings. An earlier systematic review of food insecurity among post-secondary students in any country by Bruening and colleagues [12] identified 18 peer reviewed studies and 41 studies from the grey literature, finding an average rate of food insecurity of 42% (range 12.5–84%). This review suggests that food insecurity is associated with financial independence, poor health, and adverse academic outcomes [12]. A scoping review exploring food insecurity among college students in the USA sought to develop a weighted estimated prevalence of food insecurity among students, finding that food insecurity ranged from 10 to 75% [19]. Finally, a systematic review exploring the relationship between food insecurity and dietary outcomes among post-secondary students in countries with the highest number of university students according to the World Bank identified 16 studies in the peer review literature [20]. According to this review, the prevalence of food insecurity among students ranged from 21 to 82%; lower intakes of healthy foods and higher intakes of unhealthy foods were observed in food-insecure students, and students who were food insecure were found to skip meals more often than food secure students [20].

The findings of these previous reviews underscore the importance of exploring and responding to food insecurity in student populations in order to create interventions that will best meet the needs of students and educational providers. Extending on these previous studies to incorporate a recent period of increased food insecurity and associated impacts, and to include all high-income countries, and to conduct a meta-analysis, this review synthesizes evidence from published studies that describe prevalence of food insecurity among students in post-secondary educational institutions. The findings of this review may be used to guide changes in the ways that post-secondary educational institutions support and provide assistance to students.

## Method

### Protocol and Registration

This review adheres to guidelines outlined in the Preferred Reporting Items for Systematic Reviews and Meta-Analyses (PRISMA) statement [21] (see Supplementray file 4). The review protocol was prospectively submitted to the International Prospective Register of Systematic Reviews (PROSPERO) for registration (PROSPERO identifier: CRD42023468944).

### Eligibility Criteria

Included studies were those that included an assessment of food insecurity among students attending post-secondary institutions. Studies that described the screening tools they used were considered as were studies that incorporated food insecurity screening as part of a multidomain screening tool. Studies that were conducted with students attending a post-secondary institution in any World Bank high income country were eligible [22]. Studies were excluded if the food insecurity assessment was not reported or if the students were not attending a post-secondary educational institution.

### Search Strategy and Sources

A systematic search of four electronic databases, Medline, CINAHL, Global Health, and Embase was undertaken in November 2023. Key thematic areas searched were food insecurity and education (see Table 1 for details of search terms). To gain a comprehensive collection of all published articles that report on any examination of food insecurity among post-secondary students, no temporal limitations were placed on the search. Only English language peer-reviewed articles were considered. Four authors independently reviewed all articles to identify relevant studies. Articles were imported into Covidence, a web-based systematic review management package [23]; duplicates were identified and removed. Articles underwent a three-step selection process. Articles were first screened by title and abstract based on the inclusion and exclusion criteria outlined above. Any article that clearly did not meet the inclusion criteria was removed at this stage; any that did or possibly could meet the inclusion criteria were retained. The full text of the remaining articles were obtained for further assessment. At least two authors independently read all remaining articles to determine whether the article met the inclusion criteria. Any articles at this stage that clearly did not meet the inclusion criteria were removed, and disagreements were discussed and settled by consensus between authors.

**Table 1:** PICOS criteria for inclusion of studies

Parameter	Criterion
Population	Human subjects who were students at an institution of higher education (search terms: “university student*” “tertiary student*” “college student*” “post-secondary student*” “higher education student*” “TAFE student*” “undergrad* student*”)
Intervention/exposure	Food insecurity (search terms: “food insecur*” “food secur*” “food availab*” “food insufficien*” “food sufficien*” “food access” “food stability*” “food utili? ation” “food bank” “food pantr*” “food poverty” “hunger” “poverty”)
Control/comparator	Non-food insecure students
Outcome	Food insecurity
Study design	Peer-reviewed human studies of any design

### Data Extraction and Analysis

Data, including key characteristics of the study, location, population characteristics, food security status, data collection method, sample size, and primary and secondary outcomes, were extracted into a standardised table for analysis. This allowed reviewers to draw common themes from the data. The pooled prevalence of food insecurity was obtained using a generalised linear random effects model with a logit link function. Subgroup analysis was performed by the main food insecurity measurement tools. Heterogeneity among studies was assessed using I^2^ (> 75%, 25–75% and < 25% for substantial, moderate and low heterogeneity respectively) and Cochran’s Q chi-squared test. Potential differences in food insecurity prevalence by sex distribution and mean age of the study participants, study year (proxied by mid-study year) were tested in a meta-regression using a similar generalised linear random effects model as above, with the potential source of heterogeneity as a single predictor in three separate models. The meta-analysis was done in R version 4.4.1, using the package meta.

## Results

**Fig. 1:**
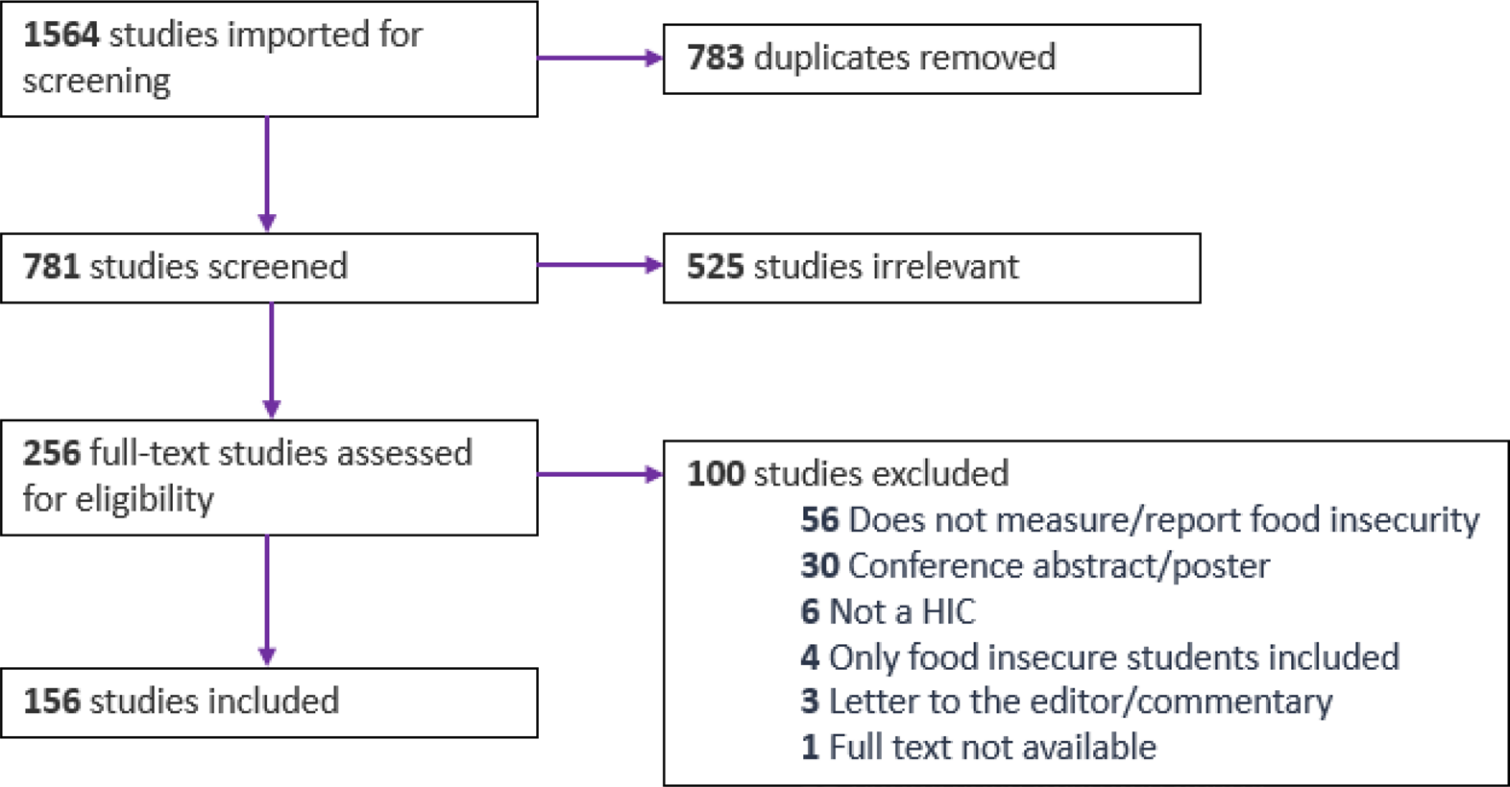
PRISMA flow diagram

Following the exclusion of 783 duplicates, 781 articles were assessed against the eligibility criteria. Title and abstract screening resulted in the exclusion of 525 articles, leaving 256 articles for full-text review. Full text review resulted in 156 studies included in this review (see Supplementary file 1 for a list of all included studies), excluded studies and reasons are listed in Supplementary file 2. See Fig. 1 for an overview of the selection process.

**Fig. 2:**
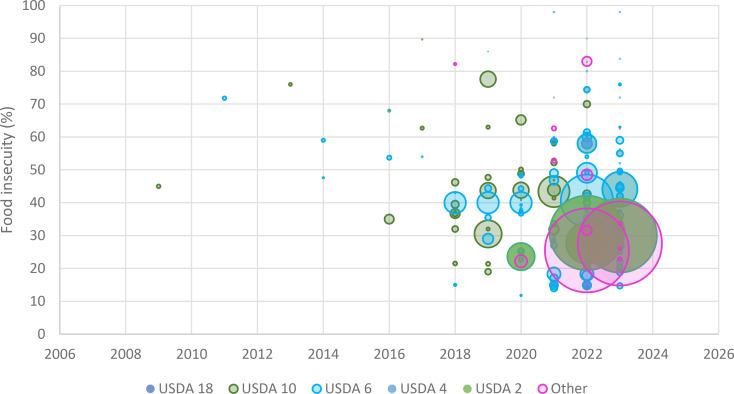
Publication year, sample size, food insecurity measure, and food insecurity estimate

The majority (*n* = 131, 83.9%) of studies were cross-sectional, eleven studies were qualitative, six were cohort studies, and the remaining eight were non-randomised or non-experimental studies. Sample sizes ranged from 10 to 122,269 participants (total participants 743,075; 409,047 women) with a median of 4764. Most studies (*n* = 153, 98%) reported how they collected food security data, with most (*n* = 134, 85.9%) employing an online survey. No temporal limitations were imposed on the search; however, all articles were published after 2009, with more than three quarters (*n* = 121, 77.5%) published after 2020. Figure 2 demonstrates this recent increase in publication of studies by displaying each study alongside its final publication year, sample size, and food insecurity measure and estimate.

Most studies (*n* = 139, 88%) were conducted with students in post-secondary institutions in the USA, followed by studies conducted in Australia (*n* = 8, 5%), Canada (*n* = 4, 3%), France (*n* = 2, 1%), with one each conducted in Germany, Greece, and Spain. Only 24 of the 156 studies included the food insecurity status of international students, of which 60% (14/24) were conducted in the USA, 30% (7/24) were conducted in Australia and the remaining 3 were conducted in Canada. One hundred (64%) studies reported the mean age of participants; across these studies the average age was 22.4 years (range 18 to 78 years). In most studies that reported age, the mean age was over 22 years (*n* = 72, 46%). Most studies reported the sex of the participants (*n* = 142, 91%) and in most of these (*n* = 137, 87.8%), women comprised half or more of the participants. See Table 2 for an overview of the study characteristics and Supplementary file 1 for a full description of each study.

**Table 2:** General characteristics of the sample (*n* = 156)

General characteristics	Studies *n* (%)
Study location	
USA Australia Canada France Germany Greece Spain	139 (88) 8 (5) 4 (3) 2 (1) 1 (1) 1 (1) 1 (1)
Food insecurity measurement tool	
USDA^*^ 18 USDA 10 USDA 6 USDA 4 USDA 2 HFIAS^^^ HVS^$^ Other	6 (4) 56 (36) 72 (46) 1 (1) 8 (5) 3 (2) 4 (2) 6 (4)
Food insecurity measurement period	
1 month/30 days 12 months Other period Not reported	30 (20) 65 (42) 18 (11) 43 (27)
Mean Age	
18 19 20 21+ Not reported	4 (2) 9 (6) 15 (10) 72 (46) 56 (36)

^*^United States Department of Agriculture Household Food Security Survey Module

^^^ Household Food Insecurity Access Scale

^$^ Hunger Vital Sign

### Food Insecurity

The pooled estimate for food insecurity was 42.2%, (95% CI = 38.8–45.8%), with substantial heterogeneity among studies (I^2^ = 99.4%, Cochran’s Q chi-squared test *p* < 0.001, as shown in the forest plot in Supplementary File 4). Pooled food insecurity prevalences were similar using different measurement tools (Table 3). The meta-regression analysis showed that mean age of participants partially explained heterogeneity among studies (*n* = 100 studies, increase of 9.1% (95% CI 1.6–16.6%) in food insecurity prevalence per year of age, *p* = 0.017) and possibly by year of study (*n* = 141 studies, decrease of 5.1% (95% CI − 0.7–10.8%) per year, *p* = 0.082), while sex (*n* = 143 studies, increase of 0.6% (95% CI − 0.9–2.1%) in food insecurity prevalence per % increase in proportion of female participants, *p* = 0.428) was not a significant factor.

**Table 3:** Pooled estimate of food insecurity prevalence by measurement tools

Food insecurity measurement tool	Food insecurity prevalence (95% CI)	I^2^	*p*-value*
USDA^^^ 18 (*n* = 6)	39.9% (17.7–67.2%)	98.7%	< 0.001
USDA 10 (*n* = 56)	43.1% (37.8–48.5%)	99.2%	< 0.001
USDA 6 (*n* = 72)	40.8% (36.2–45.5%)	98.4%	< 0.001
USDA 2 (*n* = 8)	40.0% (25.1–57.0%)	98.9%	< 0.001
HFIAS^&^/HVS^$^/Other (*n* = 15)	47.6% (35.8–59.7%)	99.7%	< 0.001

* Cochran’s Q chi-squared test for heterogeneity

^^^ United States Department of Agriculture Household Food Security Survey Module

^$^ Hunger Vital Sign

^&^ Household Food Insecurity Access Scale

Almost all studies employed one form of the United States Department of Agriculture (USDA) household food security survey module [24]. 72 studies used the USDA-6 item tool, 56 used the USDA-10 item tool, 8 used the USDA-2 item tool, 6 used the USDA-18 item tool, and just one used each the USDA-4 and USDA-3 item tool. The remaining 13 studies employed a less common tool including the Hunger Vital Sign (HVS) [25], the Household Food Insecurity Access Scale (HFIAS) [26], or a single item measure [27]. One study used both the USDA-6 item and the single item measure.

**Fig. 3:**
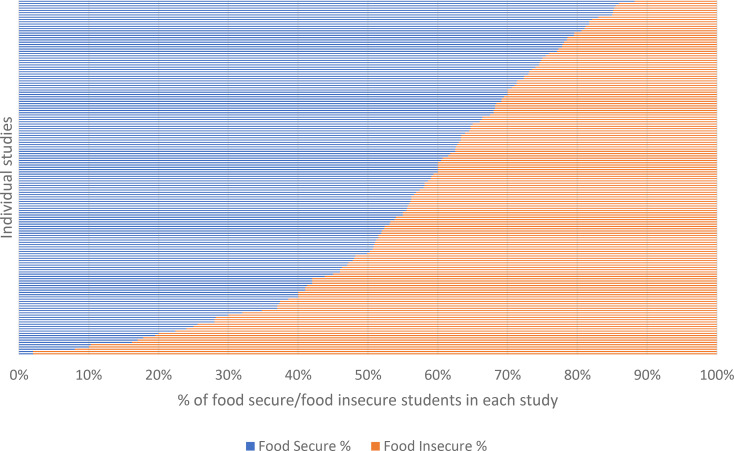
Food security across all studies

Food insecurity across the whole sample ranged from 11.8 to 98% (see Fig. 3 for a representation of food insecurity status across the whole sample). Of the 114 studies that reported the reference period for food security, 63 used a 12-month reference period, and 31 used a 1 month or 30-day reference period. The remaining studies used a reference period specific to the study, for example related to the Covid pandemic, the academic year, or some other reference period. Studies that employed the 12-month reference period identified an average food insecurity prevalence of 42.5% (SD 17.28, range 14.7–98%). Studies that employed the 1 month or 30-day reference period identified an average food insecurity prevalence of 46.74% (20.63, range 14-89.7%).

Recruitment inclusion criteria varied across studies. Sixteen of the 156 studies (10.3%) only recruited students who were considered at risk or likely to be more risk of food insecurity than the general student population. Eleven studies recruited only students who had received food aid from their institution; among these studies, the average prevalence of food insecurity was 70.0% (SD 21, range 30–98). Three studies recruited all or mostly students who screened at risk of food insecurity prior to participation; the range of food insecurity in these studies was 80-98%. A further two studies recruited only students who screened as low income, and these studies reported a food insecurity prevalence of 45% and 58.7%.

When considered by country, food insecurity was highest in the USA (53.5%, SD 24.9, 139 studies), followed by Canada (48.4%, SD 28.1, 4 studies) and Australia (44.3%, SD 14.3, 8 studies). Average food insecurity across all studies that were conducted during or after the Covid pandemic was 36.6% (SD 17.7, 46 studies), while those that collected data before the pandemic (prior to March 2020) recorded an average prevalence of food insecurity of 45.1% (SD 19, 89 studies); 21 studies did not report when data collection occurred.

### Academic Outcomes and Food Insecurity

Of the 156 studies, 38 (24%) reported a relationship between food security status and academic performance and/or academic outcomes. All 38 studies were conducted in North America (36 in USA, 2 in Canada), 24 reported on the relationship between grade point average (GPA) and food security status, with most (*n* = 22/24, 92%) reporting that food insecurity was associated with a lower GPA. Of the 14 studies that reported the impact of food insecurity on academic performance or outcomes without reporting GPA, 5 studies used another measure (for example, withdrawal from studies) and 9 described the self-reported negative impacts of food insecurity on academic performance.

### Health Outcomes and Food Insecurity

Fewer than half of studies (*n* = 65/156, 42%) reported on one or more health outcomes in relation to food insecurity. Most studies (*n* = 60/65, 92%) were conducted in North America (57 in US, 3 in Canada), four were conducted in Australia, and one in Germany. The association between poorer physical health/overall health and food insecurity was reported in 17 (26%) of the 65 studies, while findings of the association between poor mental health outcomes and food insecurity (*n* = 42, 65%), including depression (*n* = 18), anxiety (*n* = 13), psychological or mental distress (*n* = 8), stress (*n* = 5), loneliness (*n* = 5), poor sleep quality (*n* = 5), reduced wellbeing (*n* = 4), suicide behaviours (*n* = 2), and psychotic experiences (*n* = 1) were also found. Almost one third of studies (*n* = 20/65, 31%) reported a relationship between obesity, diet quality and/or eating behaviours and food insecurity. Ten studies found that obesity, overweight and/or a higher BMI were associated with food insecurity and one study found obesity/overweight was not associated with food insecurity. Five studies found eating disorders or disordered eating behaviours were associated with food insecurity, and four studies found a relationship between low quality diet and/or obesogenic behaviours and food insecurity.

## Discussion

Food insecurity among students attending post-secondary institutions has gained increasing attention as cost-of-living pressures in the post-pandemic period impact large sections of society. To understand the extent of the problem, this study sought to systematically search the peer reviewed literature describing food insecurity among students attending post-secondary institutions to explore the prevalence of food insecurity, the measurement tools used, and to understand the outcomes of food insecurity when studying.

A key aim of this review was to understand the prevalence of food insecurity among post-secondary students. Across the 156 studies, the pooled estimate for food insecurity was 42.7%, (95% CI = 39.3–46.2%), with substantial heterogeneity; food insecurity increases by age of participant but likely decreases over time. This is much higher than the prevalence of food insecurity in the general community in the countries from which the studies were conducted: for example, in the USA population level food insecurity is 12.8% [28], in Australia it is between 4% and 13% [29], while in Canada it is 16.9% [30]. The estimate of food insecurity in this study is also higher than other populations groups. For example, a systematic review exploring the relationship between severe mental illness and food insecurity found a prevalence of food insecurity of 41% [31], a study exploring food insecurity among households with infants found household food insecurity was 12.5% [32], while a global estimate of food insecurity for households with children under 15 years is 41% [33]. The results of the current study suggest that students attending post-secondary institutions are experiencing or are at risk of experiencing high rates of food insecurity, a situation that can have potential negative implications on their studies and health outcomes.

The health implications of food insecurity have been well documented. Among children, food insecurity is associated with increased risks of some birth defects [34], anaemia [35], poor nutrient intake [36], cognitive problems [37] poor mental health and anxiety [38], poorer general health [39–42], with having asthma [39], behavioural problems [43], depression [38, 42], suicide ideation [42, 44], and worse oral health [45, 46]. Among adults, food insecurity is related to poor nutrition [47], poor mental health [48–50], chronic disease [51, 52], limited health care utilisation [53, 54], poor or fair health [55], and poor sleep [56]. The drivers of household food insecurity in high income countries are complex and include financial instability or insecurity [8, 57, 58], inequality and racism [8, 59], climate shocks [60], housing instability and social risk factors [61], and disability [62]. This review and meta-analysis identified a small number of studies that explored the relationships between food insecurity and health outcomes, finding that those students who were food insecure were more likely to have poor health outcomes. Consistent with the existing literature on the impact of food insecurity on health, poor health outcomes identified here included the impact on nutrition choices, disability, self-reporting fair or poor physical health, depression, anxiety and other symptoms of psychological distress, poor sleep, and limited access to health care. Given the limited number of studies that explored these health and wellbeing outcomes, 65 of 156, and the often self-reported nature of the data collection, novel, innovative, and objective ways to assess the health outcomes of food insecurity on physical and mental health outcomes are needed [63].

This review also identified a number of studies that describe the relationship between food insecurity and academic outcomes and/or performance. Findings indicate that those students who were food insecure, were more likely to have lower GPA than those who were food secure. This is consistent with the results of a systematic review that explored academic outcomes in children who live in food insecure households, where there was a dose response between increasing food insecurity and poorer academic outcomes [43]. Poorer academic performances have been found in food-insecure students, with some studies suggesting that students are forced to withdraw from their studies as a result of food insecurity-related poor academic performance [13, 64], while another review suggests that absenteeism is an outcome of food insecurity among students [65]. Limiting the conclusions able to be drawn from this review is that only a small proportion of studies reviewed reported on these outcomes, just 38 of 156 studies, and all studies were located in North America. As such, more research is needed on the relationship between academic outcomes and food insecurity and food insecurity and physical and mental health outcomes specifically among students attending post-secondary institutions.

Only 24 of the 156 studies included the food insecurity status of international students. Other research suggests that international students experience financial insecurity [66], are vulnerable to exploitation, and often face health challenges that they struggle to address [67]. The small number of studies included in this review that explored the experiences of food insecurity among international students suggest that international students may experience food insecurity at a higher rate than domestic students. This is consistent with a previous scoping review exploring food insecurity and nutrition among international students which suggests that international students may be more vulnerable to food insecurity [68]. With the number of international students increasing in many high income countries, there is a need for further exploration of food insecurity among this population group.

Solutions to postsecondary student food insecurity are emerging, but it is still in its infancy. A recent systematic review exploring the impact of interventions that seek to address food insecurity on university campuses found only eight eligible studies [69]. Most of these studies described food pantries and food voucher programs, also identified were nutrition education programs and programs that sought to improve diet quality. Like interventions in other contexts [11, 70], the findings of this review of interventions identified that programs that were comprised of multiple ways to respond to food insecurity were those that were most successful at promoting food security [69]. This is consistent with other reviews that identified community and system level solutions to be more effective than food pantry solutions alone [71, 72]. Taken together, this previous research demonstrates that solutions to food insecurity exist. While solutions that prioritise food pantries or the distribution of food may only provide a short term or emergency response, other more integrated and multicomponent solutions may have potential for long term success. These multicomponent solutions need to be considered in any response to food insecurity on university campuses.

### Limitations

There are some limitations of this review that need to be acknowledged. Given the large number of studies included in this review, we did not conduct an evaluation of the quality of the studies, as such it is possible that there is a range in the quality of the research conducted and presented here. Only English language studies were included in the review; it is possible that other high-income countries are experiencing increasing food insecurity among their student populations and these studies have not been captured here. Most of the studies included in this review were conducted in the USA, making the results challenging to generalise to other countries. Finally, it is possible that the estimate of food insecurity presented here is an overrepresentation of the problem of food insecurity as these are largely convenience samples, with some studies sampling only recipients of food aid.

## Conclusion

Despite the clear findings of this review, there are a number of questions that remain unanswered and are avenues for future research. Firstly, there needs to be a deeper exploration of why food insecurity among this group is so high; for example, is it related to their status as a student or were they experiencing food insecurity before arriving on campus. Secondly, there needs to be more exploration on the variation among different student groups. While some of the reviewed studies reported the food insecurity prevalence of international and domestic students, more work needs to investigate the experiences of these groups, as well as first in family/first-generation students, postgraduate versus undergraduate students, and students who have arrived on campus from more disadvantaged areas. Finally, more research attention needs to be paid to the impact of food insecurity on student success and retention, and their experiences post-study. While some studies explored academic outcomes, more long-term research exploring the impact of food insecurity on the student experience will assist postsecondary institutions in creating interventions to address student food insecurity.

## Key References

- Clapp, J., et al., The case for a six-dimensional food security framework. Food Policy, 2022. 106: p. 102,164.
-
  - This article sets out the rational for the expansion of the pillars to food security to include an additional two pillars: sustainability and agency. The six-dimensional food security framework allows consideration for people to exercise control over their lives.
- Gallegos, D., et al., Food insecurity and child development: a state-of-the-art review. International Journal of Environmental Research and public health, 2021. 18(17): p. 8990.
-
  - This important state-of-the-art review explores the research indicating household food insecurity impedes children from reaching their full physical, cognitive, and psychosocial potential. This review describes the impact of the severity and persistence of food insecurity on child development; examines the proximal and distal factors which may interplay; and outlines directions for future research.
- Elgar, F.J., et al., *Relative food insecurity*,* mental health and wellbeing in 160 countries.* Social Science & Medicine, 2021. **268**: p. 113,556.
-
  - This study explores the concept of relative food insecurity as it relates to mental health, lower positive wellbeing, and lower life satisfaction. The analysis found that relative food insecurity was related to mental health and wellbeing where the prevalence of food insecurity was lower. The findings underscore the negative health consequences of material deprivation and unfavourable social comparisons.

## Electronic Supplementary Material

Below is the link to the electronic supplementary material.

Supplementary Material 1

Supplementary Material 2

Supplementary Material 3

Supplementary Material 4

## Data Availability

No datasets were generated or analysed during the current study.
