# Supplementary material for: Food Insecurity Among Post-Secondary Students in High Income Countries: Systematic Review and Meta-Analysis
Source: Curr Nutr Rep. 2025 Apr 8;14(1):58. doi: 10.1007/s13668-025-00651-2 (PMC11978725; doi:10.1007/s13668-025-00651-2)
Supplement: Supplementary file 2 — Supplementary Material 2 [file 13668_2025_651_MOESM2_ESM.docx]

**Supplementry file 2. Excluded studies**

1. Ahmad NSS, Sulaiman N, Sabri MF. Food Insecurity: Is It a Threat to University Students' Well-Being and Success? *International journal of environmental research and public health* 2021; **18**(11).

Reason for exclusion: Not a HIC

2. Ahmad NSS, Sulaiman N, Sabri MF. Psychosocial Factors as Mediator to Food Security Status and Academic Performance among University Students. *International journal of environmental research and public health* 2022; **19**(9).

Reason for exclusion: Not a HIC

3. Ahmed T, Ilieva RT, Clarke A, Wong H. Impact of a student-led food insecurity intervention on diverse community college students. *Journal of Hunger & Environmental Nutrition* 2023; **18**(1): 112-22.

Reason for exclusion: Does not measure/report food insecurity

4. Aldubaybi AA, Coneyworth LJ, Jethwa PH. The prevalence of food insecurity among UK university students. *Proceedings of the Nutrition Society* 2022; **81**(Oce5): 1-.

Reason for exclusion: Conference abstract/poster

5. Alston L, Powell K, O'Briant J, et al. Food Insecurity and Campus Food Pantry Use Among College Students During and Post the COVID-19 Pandemic: A Qualitative Study. *Current developments in nutrition* 2023; **7**.

Reason for exclusion: Conference abstract/poster

6. Anderson A, Lazarus J, Anderson Steeves E. Navigating Hidden Hunger: An Exploratory Analysis of the Lived Experience of Food Insecurity among College Students. *International journal of environmental research and public health* 2022; **19**(19).

Reason for exclusion: Only food insecure students included

7. Anziano J, Zigmont VA. Understanding food insecurity among college athletes: A qualitative study at a public university in New England. *Journal of athletic training* 2023.

Reason for exclusion: Only food insecure students included

8. Armstrong DB, Gillespie DR, King M, Collins A. Filthy fridges, food security, and freegans: food-related behaviours of students in the UK. *Appetite* 2023; **189**.

Reason for exclusion: Conference abstract/poster

9. Barr M, McNamara J. Interactive Nutrition Literacy Related to Food Insecurity Status and Self-Perceived Health Among College Students. *Current developments in nutrition* 2023; **7**.

Reason for exclusion: Conference abstract/poster

10. Bauch C, Torheim LE, Almendingen K, Molin M, Terragni L. Food Habits and Forms of Food Insecurity among International University Students in Oslo: A Qualitative Study. *International journal of environmental research and public health* 2023; **20**(3).

Reason for exclusion: Does not measure/report food insecurity

11. Becerra MB, Bol BS, Granados R, Hassija C. Sleepless in school: The role of social determinants of sleep health among college students. *Journal of American college health : J of ACH* 2020; **68**(2): 185-91.

Reason for exclusion: Does not measure/report food insecurity

12. Blennerhassett C, Peacock M, Clayton S. Does term-time employment protect students from food insecurity in UK Higher Education Institutions? *South African Journal of Clinical Nutrition* 2021; **34**(3): 32.

Reason for exclusion: Conference abstract/poster

13. Brown NI, Buro AW, Jones R, et al. Multi-Level Determinants of Food Insecurity among Racially and Ethnically Diverse College Students. *Nutrients* 2023; **15**(18).

Reason for exclusion: Does not measure/report food insecurity

14. Bydalek K, Williams SG, Fruh SM, et al. Food insecurity among nursing students: A mixed methods study. *Nursing forum* 2020; **55**(4): 547-52.

Reason for exclusion: Does not measure/report food insecurity

15. Cedillo Y, Harman T, Davis E, Durham L, Fernandez J, Smith D. The Effect of Food Security, Psychological Well-being, and Stress on BMI and Diet-related Behaviors. *Obesity* 2022; **30**: 160-1.

Reason for exclusion: Conference abstract/poster

16. Conrad AG, Tolar-Peterson T, Gardner AJ, Wei T, Evans MW, Jr. Addressing Food Insecurity: A Qualitative Study of Undergraduate Students' Perceptions of Food Access Resources. *Nutrients* 2022; **14**(17).

Reason for exclusion: Does not measure/report food insecurity

17. Correia KM, Bierma SR, Houston SD, et al. Education Racial and Gender Disparities in COVID-19 Worry, Stress, and Food Insecurities across Undergraduate Biology Students at a Southeastern University. *Journal of microbiology & biology education* 2022; **23**(1).

Reason for exclusion: Does not measure/report food insecurity

18. Davidson A, Morrell J. Food insecurity among undergraduate students. *FASEB Journal* 2015; **29**(1).

Reason for exclusion: Conference abstract/poster

19. Davis H, Sisson SB, Clifton S. A call for evidence to support food security interventions on college campuses. *Journal of American college health : J of ACH* 2021; **69**(6): 693-5.

Reason for exclusion: Does not measure/report food insecurity

20. Ehmke MD, Katare B, Kiesel K, Bergtold JS, Penn JM, Boys KA. U.S. agricultural university students' mental well-being and resilience during the first wave of COVID-19: Discordant expectations and experiences across genders. *Applied economic perspectives and policy* 2022; **44**(1): 129-61.

Reason for exclusion: Does not measure/report food insecurity

21. El Zein A, Shelnutt K, Colby S, et al. Socio-demographic Correlates and Predictors of Food Insecurity among First Year College Students. *Journal of the Academy of Nutrition & Dietetics* 2017; **117**(10): A146-A.

Reason for exclusion: Conference abstract/poster

22. Ellis A, Burns T, Buzzard J, Dolan L, Register S, Crowe-White K. Food Insecurity among College Students Does Not differ by Affiliation in Greek Life. *Journal of the Academy of Nutrition & Dietetics* 2017; **117**(10): A145-A.

Reason for exclusion: Conference abstract/poster

23. Enriquez JP, Ader D. Examining food security among minority student groups, with particular attention on diversity and inclusion. *Journal of American college health : J of ACH* 2023: 1-8.

Reason for exclusion: Does not measure/report food insecurity

24. Fortin K, Harvey S, Swearingen White S. Hidden Hunger: Understanding the Complexity of Food Insecurity Among College Students. *Journal of the American College of Nutrition* 2021; **40**(3): 242-52.

Reason for exclusion: Does not measure/report food insecurity

25. Frank LB. "Free food on campus!": Using instructional technology to reduce university food waste and student food insecurity. *Journal of American college health : J of ACH* 2022; **70**(7): 1959-63.

Reason for exclusion: Does not measure/report food insecurity

26. Frank LB, Finkbinder EM, Powell VS. "Free food on campus!": a novel use of instructional technology to reduce university food waste and feed hungry students. *Journal of Hunger & Environmental Nutrition* 2021; **16**(5): 706-24.

Reason for exclusion: Does not measure/report food insecurity

27. Freire AN, Fleischer SH, Brown KN, et al. Article prevalence of female athlete triad risk factors among female international volunteers and college age-matched controls. *International journal of environmental research and public health* 2022; **19**(3).

Reason for exclusion: Does not measure/report food insecurity

28. Freudenberg N, Goldrick-Rab S, Poppendieck J. College Students and SNAP: The New Face of Food Insecurity in the United States. *American journal of public health* 2019; **109**(12): 1652-8.

Reason for exclusion: Does not measure/report food insecurity

29. Frymark EE, Stickford JL, Farris AR. A Nutritional and Environmental Analysis of Local Food Pantries Accessible to College Students in Rural North Carolina. *Journal of Appalachian health* 2020; **2**(2): 24-35.

Reason for exclusion: Does not measure/report food insecurity

30. Gaines A, Knol LL, Robb CA, Sickler SM. Food Insecurity Is Related to Cooking Self-Efficacy and Perceived Food Preparation Resources among College Students. *Journal of the Academy of Nutrition & Dietetics* 2012; **112**: A11-A.

Reason for exclusion: Conference abstract/poster

31. Gamba RJ, Schmeltz MT, Ortiz N, et al. 'Spending all this time stressing and worrying and calculating': marginal food security and student life at a Diverse Urban University. *Public health nutrition* 2021; **24**(10): 2788-97.

Reason for exclusion: Does not measure/report food insecurity

32. Gamba RJ, Wood LM, Ampil A, et al. Investigating the Feasibility of a Restaurant Delivery Service to Improve Food Security among College Students Experiencing Marginal Food Security, a Head-to-Head Trial with Grocery Store Gift Cards. *International journal of environmental research and public health* 2021; **18**(18).

Reason for exclusion: Does not measure/report food insecurity

33. Gande S, Mangal RK, Stead TS, Ganti L. State of nutrition amongst US college students: dataset of a national survey study. *BMC research notes* 2023; **16**(1): 10.

Reason for exclusion: Does not measure/report food insecurity

34. Goldrick-Rab S, Coca V, Gill J, Peele M, Clark K, Looker E. Self-reported COVID-19 infection and implications for mental health and food insecurity among American college students. *Proceedings of the National Academy of Sciences of the United States of America* 2022; **119**(7).

Reason for exclusion: Does not measure/report food insecurity

35. Goon S, Slotnick M, Leung C. Subjective Social Status and Its Relationship To Diet and Health Behavior Among Students at a Large, Midwestern University. *Current developments in nutrition* 2023; **7**.

Reason for exclusion: Conference abstract/poster

36. Guerithault N, McClure SM, Ojinnaka CO, Braden BB, Bruening M. Resting-State Functional Connectivity Differences in College Students with and without Food Insecurity. *Nutrients* 2022; **14**(10).

Reason for exclusion: Does not measure/report food insecurity

37. Hafiz AA, Gallagher AM, Hill AJ. A qualitative study to explore the experiences of university students which influence eating behaviours when living away from home. *Proceedings of the Nutrition Society* 2016; **75**(OCE3): E194.

Reason for exclusion: Conference abstract/poster

38. Hagedorn RL, Barr ML, Famodu OA, Morris AM, Clark RL, Olfert MD. Food insecurity among college students at West Virginia University and self-reported health status. *FASEB Journal* 2017; **31**(1).

Reason for exclusion: Conference abstract/poster

39. Hanbazaza M, Ball GDC, Farmer A, Maximova K, Willows ND. Filling a need: sociodemographic and educational characteristics among student clients of a university-based campus food bank. *Journal of Hunger & Environmental Nutrition* 2016; **11**(4): 569-77.

Reason for exclusion: Does not measure/report food insecurity

40. Hardin-Fanning F. Exploration of Barriers to Use of Community Food Resources in Community College Students in Rural Appalachia. *Journal of Hunger and Environmental Nutrition* 2023.

Reason for exclusion: Does not measure/report food insecurity

41. Haskett ME, Cooke NK, Goodell LS. College Student Food and Housing Insecurity: Students’ Perceived Determinants, Consequences, and Resilience. *Journal of Hunger and Environmental Nutrition* 2023.

Reason for exclusion: Does not measure/report food insecurity

42. Hattangadi N, Vogel E, Carroll LJ, Côté P. "Everybody I know is always hungry ... but nobody asks why": university students, food insecurity and mental health. *Sustainability (2071-1050)* 2019; **11**(6): 1571.

Reason for exclusion:

43. Hattangadi N, Vogel E, Côté P, Carroll L. Exploring the prevalence and association of food insecurity and psychological distress in university students. *Canadian journal of dietetic practice and research: a publication of Dietitians of Canada = Revue canadienne de la pratique et de la recherche en diététique: une publication des Diététistes du Canada* 2018; **79**(3): 146-.

Reason for exclusion: Conference abstract/poster

44. Jeng A, McCarren EM, Suñé AJ. Food security and part-time work for students: do race and region play a role? *Journal of American college health : J of ACH* 2022: 1-9.

Reason for exclusion: Does not measure/report food insecurity

45. Kent K, Visentin D, Murray S. Food insecurity among university students, professional and academic staff at the University of Tasmania. *Proceedings of the Nutrition Society* 2023; **82**(OCE2): 1-.

Reason for exclusion: Conference abstract/poster

46. Kim Y, Murphy J, Craft K, Waters L, Gooden BI. "It's just a constant concern in the back of my mind": Lived experiences of college food insecurity. *Journal of American college health : J of ACH* 2022: 1-8.

Reason for exclusion: Does not measure/report food insecurity

47. Kirby SR, Linde JA. Understanding the Nutritional Needs of Transgender and Gender-Nonconforming Students at a Large Public Midwestern University. *Transgender health* 2020; **5**(1): 33-41.

Reason for exclusion: Does not measure/report food insecurity

48. Kozak B, Tipsword-Kizer A, Lanier J. Combating Student Food Insecurity: An Examination of the Usage and Outreach of a College Campus Food Pantry. *Health Education Monograph Series* 2019; **36**(1): 32-5.

Reason for exclusion: Conference abstract/poster

49. Lederer AM, Hoban MT, Lipson SK, Zhou S, Eisenberg D. More Than Inconvenienced: The Unique Needs of U.S. College Students During the COVID-19 Pandemic. *Health education & behavior : the official publication of the Society for Public Health Education* 2021; **48**(1): 14-9.

Reason for exclusion: Does not measure/report food insecurity

50. Lee S, Ball GDC, Farmer A, Willows ND. Exploring the experience of food insecurity among university students caring for children: a qualitative descriptive study. *Journal of Hunger & Environmental Nutrition* 2020; **15**(3): 360-71.

Reason for exclusion: Does not measure/report food insecurity

51. Lee Y, Yoon H, Kim T, Jung H. Food Insecurity during the Pandemic in South Korea: The Effects of University Students' Perceived Food Insecurity on Psychological Well-Being, Self-Efficacy, and Life Satisfaction. *Foods (Basel, Switzerland)* 2023; **12**(18).

Reason for exclusion: Not a HIC

52. Lemp H, Lanier J, Wodika A, Schalasky G. Impact of food insecurity on the health and well-being of college students. *Journal of American college health : J of ACH* 2023: 1-10.

Reason for exclusion: Does not measure/report food insecurity

53. Levy TM, Williams Jr RD, Housman JM, Odum M. COVID-19 Stress, Food Security, and Fruit and Vegetable Consumption among University Students. *Health Education Monograph Series* 2022; **39**(1): 11-7.

Reason for exclusion: Does not measure/report food insecurity

54. Levy TM, Williams RD, Odum M, Housman JM, McDonald JD. Impact of COVID-19 stress on food insecurity and fruit and vegetable consumption among college students. *Journal of American college health : J of ACH* 2022: 1-8.

Reason for exclusion: Does not measure/report food insecurity

55. Macke C, Averitt Taylor J, Ozaki R. Food Insecure Students' Perceptions of Campus Climate: Implications for Social Work Educators and Practitioners. *Journal of evidence-based social work (2019)* 2020; **17**(2): 237-52.

Reason for exclusion: Only food insecure students included

56. Maher JP, Hevel DJ, Reifsteck EJ, Drollette ES. Physical activity is positively associated with college students' positive affect regardless of stressful life events during the COVID-19 pandemic. *Psychology of sport and exercise* 2021; **52**: 101826.

Reason for exclusion: Does not measure/report food insecurity

57. Manboard M, Johnson CM, Thornton H, Biediger-Friedman L. The HOME Study: Understanding How College Students at a Hispanic Serving Institution Coped with Food Insecurity in a Pandemic. *International journal of environmental research and public health* 2021; **18**(21).

Reason for exclusion: Only food insecure students included

58. Maneerattanasuporn T, Techakriengkrai T, Jamphon A, Riebroy Kim S, Muangtuk Mba T. Food Insecurity Situation among Undergraduate Students During the COVID-19 Pandemic. *Asia-Pacific journal of public health* 2022; **34**(2-3): 276-8.

Reason for exclusion: Not a HIC

59. Marquis M, Talbot A, Sabourin A, Riopel C. Exploring the environmental, personal and behavioural factors as determinants for university students' food behaviour. *International Journal of Consumer Studies* 2019; **43**(1): 113-22.

Reason for exclusion: Does not measure/report food insecurity

60. Mathews A, Lee C, Colee J, Shelnutt K. Food insecurity among college students: Perceptions of snap and barriers to utilization. *Obesity* 2020; **28**(SUPPL 2): 100.

Reason for exclusion: Conference abstract/poster

61. Mawer T, O'Kane G. The potential role of food gardens in addressing food security and sustainability: A pilot study from a university campus in Act, Australia. *Revista Espanola de Nutricion Humana y Dietetica* 2016; **20**: 561-2.

Reason for exclusion: Conference abstract/poster

62. McCoin M, Thompson A, Rodriguez-Jordan J. Utilization of a teaching kitchen to enhance food security in college students. *Journal of Alternative and Complementary Medicine* 2018; **24**(7): A18.

Reason for exclusion: Conference abstract/poster

63. McNamara J, Barr M. Health Behaviors and Food Access Predict Incidence of Anxiety in College Students. *Current developments in nutrition* 2023; **7**.

Reason for exclusion: Conference abstract/poster

64. Meldrum LA, Willows ND. Food insecurity in university students receiving financial aid. *Canadian journal of dietetic practice and research : a publication of Dietitians of Canada = Revue canadienne de la pratique et de la recherche en dietetique : une publication des Dietetistes du Canada* 2006; **67**(1): 43-6.

Reason for exclusion: Does not measure/report food insecurity

65. Milanaik R, Athanasian C, Lazarevic B, Kriegel E, Rapoport EP, Chow N. Barriers to Academic Performance in Distance Learning Settings Among College Students. *Pediatrics* 2022; **149**.

Reason for exclusion: Conference abstract/poster

66. Mooney G, Drake T, Vollmer RL. A Qualitative Analysis of Eating Behaviors Among Food Insecure College Students. *Journal of nutrition education and behavior* 2023; **55**(7): 531-40.

Does not measure/report food insecurity

67. Moore D, Lindsay K, Ma E, et al. Diet Quality of Full Time College Students According to Food Security Status. *Current developments in nutrition* 2023; **7**.

Reason for exclusion: Conference abstract/poster

68. Munger B, Musich A, Reeves B, et al. Adaptation of the USDA Food Security Survey Module for College Students. *Journal of the Academy of Nutrition & Dietetics* 2022; **122**(9): A57-A.

Reason for exclusion: Conference abstract/poster

69. Nikolaus CJ, Ellison B, Nickols-Richardson SM. Are estimates of food insecurity among college students accurate? Comparison of assessment protocols. *PloS one* 2019; **14**(4): e0215161.

Reason for exclusion: Does not measure/report food insecurity

70. Oh H, Susser E, Volpe VV, et al. Psychotic experiences among Black college students in the United States: The role of socioeconomic factors and discrimination. *Schizophrenia research* 2022; **248**: 198-205.

Reason for exclusion: Does not measure/report food insecurity

71. Oonorasak K, Barr M, Pennell M, et al. Evaluation of a sustainable student-led initiative on a college campus addressing food waste and food insecurity. *Journal of Agriculture, Food Systems and Community Development* 2022; **11**(4): 223-37.

Reason for exclusion: Does not measure/report food insecurity

72. Owens M, Brito-Silva F, Kirkland T, et al. Food insecurity and overweight/obesity associated with poor diet quality during COVID-19 pandemic. *Obesity* 2020; **28**(SUPPL 2): 100-1.

Reason for exclusion: Conference abstract/poster

73. Pabani N, Lordly D, Knezevic I, Williams PL. Student Engagement with Community-Based Participatory Food Security Research: Exploring Reflections through Photovoice. *Canadian journal of dietetic practice and research : a publication of Dietitians of Canada = Revue canadienne de la pratique et de la recherche en dietetique : une publication des Dietetistes du Canada* 2020; **81**(4): 210-4.

Reason for exclusion: Does not measure/report food insecurity

74. Pepetone AB, Qutub MB, Andrade LMRD, Wallace MPP, Kirkpatrick SIPRD. Food Security Status in Relation to Co-operative Enrolment Among University of Waterloo Undergraduate Students: A Cross-Sectional Analysis. *Canadian journal of dietetic practice and research : a publication of Dietitians of Canada = Revue canadienne de la pratique et de la recherche en dietetique : une publication des Dietetistes du Canada* 2023: 1-7.

Reason for exclusion: Full text not avaliable

75. Peterson N, Freidus A, Tereshenko D. Why college students Don't access resources for food insecurity: stigma and perceptions of need. *Annals of Anthropological Practice* 2022; **46**(2): 140-54.

Reason for exclusion: Does not measure/report food insecurity

76. Price CE, Reppond HA, Sampson NR, Camp JK, Thomas-Brown K. Creating a community of practice among college campus food pantry directors in Michigan. *Journal of Community Practice* 2019; **27**(1): 96-109. Reason for exclusion:

Reason for exclusion: Does not measure/report food insecurity

77. Radtke M, Chodur G, Kemp L, Medici V, Steinberg F, Scherr R. The Use of On-Campus Food Access Resources on Diet-related Biomarkers in Plasma and Skin. *Current developments in nutrition* 2023; **7**.

Reason for exclusion: Conference abstract/poster

78. Rahal D, Shaw S. Impacts of the COVID-19 Transition to Remote Instruction for University Students. *Journal of student affairs research and practice* 2023; **60**(1): 108-22.

Reason for exclusion: Does not measure/report food insecurity

79. Rambliere L, Leservoisier C, Bedo Y, et al. Major depressive disorder in post-secondary students attending foodbanks in France. *Frontiers in public health* 2023; **11**: 1177617.

Reason for exclusion: Does not measure/report food insecurity

80. Randles A. Dietary Behaviors & Perceived Nutrition Availability of Small College Student-Athletes: a Pilot Project. *Sport Journal* 2018: 1-.

Reason for exclusion: Does not measure/report food insecurity

81. Randles A. Dietary behaviors &amp; perceived nutrition availability of small college student-athletes: a pilot project. *The Sport Journal* 2018; **21**.

Reason for exclusion: Does not measure/report food insecurity

82. Rodriguez-Jordan J, Matias S, McCoin M. Utilization of a teaching kitchen within a nutrition course increased fruit and vegetable intake among college students. *Journal of Alternative and Complementary Medicine* 2020; **26**(11): A15.

Reason for exclusion: Conference abstract/poster

83. Rosenberg L, Begdache L, Bubis S, et al. Effects of Food Insecurity on Academic Motivation Amongst Undergraduate University Students. *Current developments in nutrition* 2023; **7**.

Reason for exclusion: Conference abstract/poster

84. Sani M, Ayubi E. The Prevalence of Food Security and Insecurity Among Illinois University Students: Reporting the Strength of Association...Morris LM et al. The Prevalence of Food Security and Insecurity Among Illinois University Students. J Nutr Educ Behav Jun 2016 48(6): 376-82. New York, New York: Elsevier B.V.; 2016. p. 679-.

Reason for exclusion: Letter to the editor/commentary

85. Sani M, Ayubi E. The Prevalence of Food Security and Insecurity Among Illinois University Students: Reporting the Strength of Association. *Journal of nutrition education and behavior* 2016; **48**(9): 679.

Reason for exclusion: Letter to the editor/commentary

86. Shakya E, Whetzel C, Lanza S, Na M. Association between Food Insecurity and Depressive Symptoms in College Students Before and During the COVID-19 Pandemic. *Current developments in nutrition* 2022; **6**: 948.

Reason for exclusion: Conference abstract/poster

87. Sidebottom C, Ullevig S, Cheever K, Zhang T. Effects of COVID-19 pandemic and quarantine period on physical activity and dietary habits of college-aged students. *Sports medicine and health science* 2021; **3**(4): 228-35.

Reason for exclusion: Does not measure/report food insecurity

88. Silva FB, Osborn DE, Owens MR, et al. Influence of COVID-19 Pandemic Restrictions on College Students' Dietary Quality and Experience of the Food Environment. *Nutrients* 2021; **13**(8).

Reason for exclusion: Does not measure/report food insecurity

89. Sklar E, Chodur G, Kemp L, Scherr R, Fetter D. Understanding Perceptions of Food Security Status in College Students. *Current developments in nutrition* 2023; **7**.

Reason for exclusion: Conference abstract/poster

90. Story CR, Smith EA, Harvey IS, Thareja G, Hayes J. Exploration of how emotional social support predicts food insecurity among college students. *Journal of American college health : J of ACH* 2022: 1-5.

Reason for exclusion: Does not measure/report food insecurity

91. Ukegbu P, Nwofia B, Ndudiri U, Uwakwe N, Uwaegbute A. Food Insecurity and Associated Factors Among University Students. *Food and nutrition bulletin* 2019; **40**(2): 271-81.

Reason for exclusion: Not a HIC

92. Volpe SL. Food Insecurity Among College Students. *ACSM's Health & Fitness Journal* 2019; **23**(5): 53-4.

Reason for exclusion: Letter to the editor/commentary

93. Waity JF, Huelskamp A, Hagedorn-Hatfield RL, et al. The COVID-19 pandemic and college student food access: perspectives from students at four North Carolina universities. *Journal of Hunger & Environmental Nutrition* 2023; **18**(5): 772-88.

Reason for exclusion: Does not measure/report food insecurity

94. Walker KL, Hadjistavropoulos T, Gagnon MM, MacNab YC. Development and Validation of the Hunger Sensitivity Scale (HSS) Among University Students. *Canadian Journal of Behavioural Science* 2015; **47**(1): 1-11.

Reason for exclusion: Does not measure/report food insecurity

95. Wilson H, Amirabdollahian F, Farhat G, Macdonald-Clarke C. Association between mental health and nutritional status in university students aged 18-24 years. *Proceedings of the Nutrition Society* 2020; **79**(OCE2).

Reason for exclusion: Conference abstract/poster

96. Wilson H, Neufeld HT, Anderson K, Wehkamp C, Khoury DE. Exploring indigenous undergraduate students' experiences within urban and institutional food environments. *Sustainability (2071-1050)* 2021; **13**(18).

Reason for exclusion: Does not measure/report food insecurity

97. Wright KE, Lucero JE, Ferguson JK, et al. The impact that cultural food security has on identity and well-being in the second-generation U.S. American minority college students. *Food security* 2021; **13**(3): 701-15.

Reason for exclusion: Does not measure/report food insecurity

98. Zhai Y, Du X. Trends and prevalence of suicide 2017-2021 and its association with COVID-19: Interrupted time series analysis of a national sample of college students in the United States. *Psychiatry research* 2022; **316**: 114796.

Reason for exclusion: Does not measure/report food insecurity

99. Zigmont VA, Linsmeier AM, Gallup P. Understanding the Why of College Student Food Insecurity. *Journal of Hunger and Environmental Nutrition* 2019.

Reason for exclusion: Does not measure/report food insecurity

100. Zolfaghari H, Beyrambibi B, Azimeh I, Gholamreza A, Fereshteh P. Food insecurity and depressive symptoms among university students: a cross-sectional study from Iran &lt;./article-1-250-en.pdf &gt. *Journal of Nutrition and Food Security* 2021; **6**(1): 6-13.

Reason for exclusion: Not a HIC
