# Supplementary figures and images for: Food Insecurity Among Post-Secondary Students in High Income Countries: Systematic Review and Meta-Analysis
Source: Curr Nutr Rep. 2025 Apr 8;14(1):58. doi: 10.1007/s13668-025-00651-2 (PMC11978725; doi:10.1007/s13668-025-00651-2)

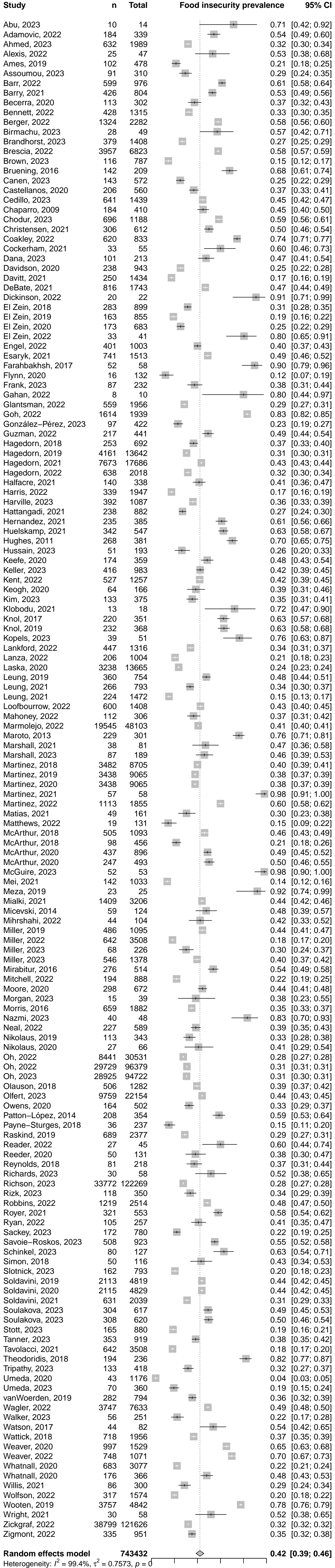

0 0.2 0.4 0.6 0.8 1

Supplement: Supplementary file 4 — Supplementary Material 4 [file 13668_2025_651_MOESM4_ESM.pdf]
